# The balance between fetal oxytocin and placental leucine aminopeptidase (P-LAP) controls human uterine contraction around labor onset

**DOI:** 10.1016/j.eurox.2023.100210

**Published:** 2023-06-21

**Authors:** Masato Yoshihara, Shigehiko Mizutani, Kunio Matsumoto, Yukio Kato, Yusuke Masuo, Arita Harumasa, Shohei Iyoshi, Sho Tano, Hidesuke Mizutani, Tomomi Kotani, Eita Mizutani, Kiyosumi Shibata, Hiroaki Kajiyama

**Affiliations:** a Department of Obstetrics & Gynecology, Nagoya University Graduate School of Medicine, Nagoya, Japan; b Nagoya University, Japan; c Daiya building Ladies Clinic, Nagoya, Japan; d Division of Tumor Dynamics and Regulation, Cancer Institute, Kanazawa University, Kanazawa, Japan; e Department of Molecular Pharmacotherapeutics, Facility of Pharmacy, Kanazawa University, Kanazawa, Japan; f Rohto Pharmaceutical Co., Ltd., Japan; g Institute for Advanced Research, Nagoya University, Nagoya, Japan; h Department of Obstetrics & Gynecology, Bantane Hospital, Fujita Health University, Nagoya, Japan

**Keywords:** Fetal oxytocin, Progesterone withdrawal, Placental leucine aminopeptidase (P-LAP), Fetal adrenocorticotropin, CYP17A in human placenta

## Abstract

A fetal pituitary hormone, oxytocin which causes uterine contractions, increases throughout gestation, and its increase reaches 10-fold from week 32 afterward. Oxytocin is, on the other hand, degraded by placental leucine aminopeptidase (P-LAP) which exists in both terminal villi and maternal blood. Maternal blood P-LAP increases with advancing gestation under the control of non-genomic effects of progesterone, which is also produced from the placenta. Progesterone is converted to estrogen by CYP17A1 localized in the fetal adrenal gland and placenta at term. The higher oxytocin concentrations in the fetus than in the mother demonstrate not only fetal oxytocin production but also its degradation and/or inhibition of leakage from fetus to mother by P-LAP. Until labor onset, the pregnant uterus is quiescent possibly due to the balance between increasing fetal oxytocin and P-LAP under control of progesterone. A close correlation exists between the feto-placental and maternal units in the placental circulation, although the blood in the two circulations does not necessarily mix. Fetal maturation results in progesterone withdrawal via the CYP17A1 activation accompanied with fetal oxytocin increase. Contribution of fetal oxytocin to labor onset has been acknowledged through the recognition that the effect of fetal oxytocin in the maternal blood is strictly regulated by its degradation by P-LAP under the control of non-genomic effects of progesterone. In all senses, the fetus necessarily takes the initiative in labor onset.

## Highlights

• Oxytocin in fetal pituitary gland increases throughout gestation.
• P-LAP/oxytocinase localized in terminal villi increases in maternal blood with advancing gestation.
• The pregnant uterus is quiescent by the balance between increasing fetal oxytocin and P-LAP in maternal blood.
• Fetal maturation results in progesterone withdrawal via the CYP17A1 activation.
• No increase in P-LAP due to progesterone withdrawal results in breakdown of the balance and uterine contraction.

## 1 Introduction

There have been massive advancements in our understanding of the normal labor onset in humans, but it is also true that much remains to be revealed. Numerous studies have reported the roles of several biochemical and hormonal factors in myometrial excitability and contractility, yet there is no clear hierarchy of events leading to labor onset. Preceding this event, the myometrium undergoes increased contracting activity, while the uterine cervix undergoes ripening to allow the passage of the fetus from the uterus. An understanding of labor onset is required for the identification of the factors controlling this hierarchy.

The pioneering work of Liggins and his colleagues in sheep first demonstrated that maturation of fetal hypothalamic-pituitary-adrenal axis triggers the labor onset and stimulates the maturation of the fetal organ systems essential for extrauterine life. Destruction of the paraventricular nucleus of the fetal hypothalamus results in the prolongation of pregnancy in sheep [1]. The human counterpart to this animal experiment is anencephaly, which is also characterized by prolonged pregnancy when women with polyhydramnios are excluded. Liggins and his colleagues have reported that a fetal signal according to fetal hypothalamic-pituitary-adrenal axis maturation contributes to labor onset in not only animals but also humans [1].

It is well known that progesterone supports pregnancy and prevents parturition by promoting myometrial quiescence. In the mid-1950 s, Csapo proposed that progesterone actively blocks labor and delivery and that parturition requires progesterone withdrawal [2]. In contrast, estrogens stimulate parturition by increasing the expression of genes that encode factors augmenting myometrial contractility and excitability. Thus, the process of parturition requires progesterone withdrawal and estrogen activation, which in most mammals are achieved by changes in circulating progesterone decrease and estrogen increase [1]. However, in humans while estrogen activation is well acknowledged [3], [4], progesterone withdrawal is not still apparent.

While the transitional zone of the fetal adrenal gland which develops in late gestation produces glucocorticoids, the fetal zone occupying the large part of the fetal adrenal cortex produces dehydroepiandrosterone (DHEA), the precursor for placental estrogen. The fetal zone of the adrenal gland expresses CYP17A1 (or P450c17) [5] which is a bifunctional enzyme with 17ɑ-hydroxylase and 17,20-lyase activities. CYP17A1 converts progesterone to 17ɑ-hydroxyprogesterone (17OHP) and then to androstenedione, and also converts pregnenolone to 17ɑ-hydroxypregnenolone and then to DHEA [6].

**Fig. 1:**
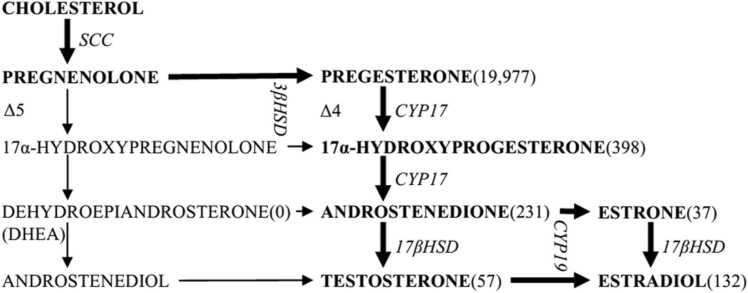
Suggested preferred steroidogenic pathway in human trophoblasts highlighted by bold letters and arrows (picograms per milligram of cellular protein produced per day) [7].

Early studies on the human placenta could not demonstrate placental activity or mRNA expression of CYP17A1. The concept subsequently emerged that the placenta was unable to convert pregnenolone or progesterone to androgen products. However, recent studies demonstrated mRNA and protein expression of this enzyme and its activity in human trophoblasts [7]. It has been shown that CYP17A1 expression in the human placenta is significantly upregulated at term [8]. The changes in pregnant uterine cervical ripening essential for delivery were shown to be associated with a significant elevation of maternal DHEA sulfate levels [9]. DHEA is converted to estrogen, which is essential in cervical softening (Fig. 1).

Oxytocin is a potent stimulant of myometrial activity and has been extensively used in clinical practice for the induction of labor. In animals circulating concentrations of this hormone are increased during labor and exhibit maximum at the time of delivery, suggesting that endogenous oxytocin may play an important role in spontaneous labor [10], [11]. Chard et al. [12] showed that fetal oxytocin is involved in the onset of human labor. Since Fekete [13] first discovered the ability of human pregnancy sera to destroy oxytocin in 1930, this hormone is known to be inactivated by oxytocinase in pregnancy sera [14]. We have cloned this enzyme and named it placental leucine aminopeptidase i.e. P-LAP. P-LAP is responsible for the degradation of both oxytocin and vasopressin [15]. Somewhat long years have passed since the epoch-making discovery of Chard et al., but oxytocin homeostasis should be reevaluated by taking into account P-LAP dynamics in maternal blood around labor onset.

In pregnancy, placental circulation occurs through two independent circulation systems: feto-placental and uterine (spiral artery)-placental lake.　Our previous study has clarified the crosstalk between the fetal vasopressin and its degrading enzyme P-LAP involved in placental circulation. We have demonstrated that in severe preeclampsia the protective roles of P-LAP break down, leading to a massive leak of fetal vasopressin into maternal circulation and consequent contraction of both maternal vessels and the uterus [16]. In this review, we will discuss the possibility of P-LAP in maternal blood being involved in labor onset. Since P-LAP is released into maternal blood from lysosomes in the villi of syncytiotrophoblasts with aid of progesterone [16], we will also discuss the dynamic aspects of P-LAP in relation to progesterone changes prior to human labor onset.

## 2 Fetal anterior pituitary hormone oxytocin

**Fig. 2:**
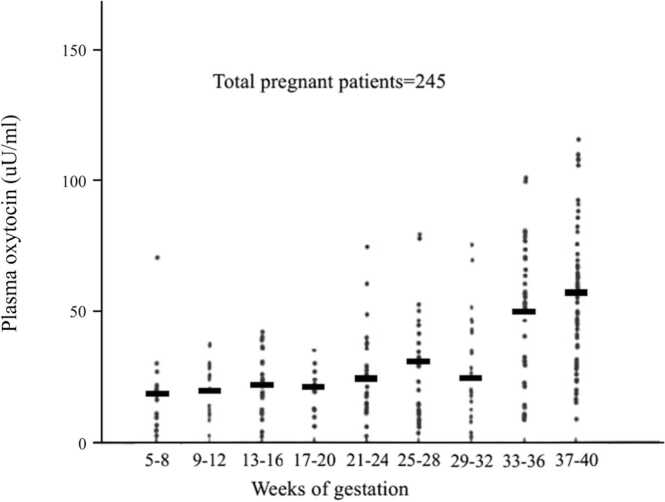
Plasma oxytocin concentrations at different stages of pregnancy. The horizontal bar represents the mean of the values at each gestational week [22].

As mentioned above, the enormous work by Liggins and his colleagues [1] has shown that the fetal hypothalamic-anterior pituitary-adrenal axis maturation is the constituent part of the hierarchy at labor onset. Chard T et al. [12] showed the release of oxytocin and vasopressin from the human fetus during labor by confirming the arterio-venous difference of cord plasma and the absence of both oxytocin and vasopressin in the cord plasma of an anencephalic fetus. At term, oxytocin levels in umbilical circulation showed higher values than in maternal circulation (1–10 pg/mL) with higher concentrations in an umbilical artery (15–40 pg/mL) than in umbilical vein (4–12 pg/mL). Such umbilical artery-vein difference suggests the active production of oxytocin from the fetal anterior pituitary gland. The positive difference of umbilical artery-vein indicates not only that the production of oxytocin by the fetus but also degradation in the placenta and/or transfer of oxytocin from fetal to the maternal circulation [17]. Fetal pituitary content of oxytocin increases throughout gestation; pituitary gland oxytocin was 10.2 ng/gland at 14–17 weeks of gestation, but its contents were 22.1–57.0 ng/gland at 32 gestational week and significantly increased to 544 ng/gland in 1- to 5-day-old term newborn infants [18]. Oosterbaan et al. [19] showed that oxytocin levels in cord blood or blood from the umbilical artery or vein after physiological labor were 65.9 pg/mL and significantly higher than those observed after elective cesarean section (∼12.9 pg/mL). These results showed that the increasing fetal oxytocin with advancing gestation is involved in normal labor [20]. Dawood et al. [21] also measured oxytocin levels in plasma during pregnancy by a modified method of Chard et al. We have also shown that the mean level of maternal plasma oxytocin gradually increases with advancing pregnancy, culminating in a marked increase during the last two months as in case of the previous report by Dawood et al. [22]. Basal levels of oxytocin increased 3–4 folds during pregnancy, and pulses of oxytocin occurred with increasing frequency, duration, and amplitude from late pregnancy throughout labor [22], [23] (Fig. 2).

## 3 The feto-maternal barrier for oxytocin

The placental circulation demonstrated a close relationship between the two circulation systems: feto-placental and maternal circulation. The terminal villi of the placenta are feto-maternal exchanging sites, but no intermingling between the maternal and fetal blood occurs in the placenta. The fetal-maternal barrier maintains maternal homeostasis by regulating the movement of fetal bioactive molecules, which are essential for fetal growth and shows the difference in their concentrations between the two circulation systems across the endothelium of the placental villi capillaries. P-LAP is localized in the syncytiotrophoblasts at the villus wall and prevents an inflow of oxytocin into the maternal blood. P-LAP was cloned as an aminopeptidase that degrades oxytocin and vasopressin [15]. Importantly, P-LAP is identical to the insulin-regulated membrane aminopeptidase (IRAP), originally identified in the fat and muscular tissues as a major protein localized in the intracellular vesicles that harbor the insulin-responsive glucose transporter type 4. Therefore, P-LAP has also been described as IRAP or IRAP/PLAP. Since P-LAP is localized in the syncytiotrophoblasts in the placenta [24], [25], a substantial clearance of oxytocin and vasopressin may occur across the fetoplacental circulation, in which P-LAP degrades these hormones in the villus tissue to control their leakage into the maternal blood. Malek et al. was not positive for the involvement of oxytocinase/ aminopeptidases in human placenta with the transfer of fetal oxytocin to the maternal circulation [17].

We have shown that placental particulate fraction actively degraded oxytocin in minutes almost completely into its constituent amino acids and amide. In addition, P-LAP liberated actively Tyr, Ile, Gln, and Asn from the cyclic structure of oxytocin. Splitting the N-terminal peptide bond by P-LAP meaning the loss of uterine action of oxytocin, was initially detected in the blood during pregnancy. We have also showed that the combination of the post-proline endopeptidase with aminopeptidase N in placenta actively liberated all amino acids and amides from oxytocin [26]. It is evident that vasopressin is degraded by human placenta [27].

## 4 P-LAP in lysosomes and extracellular spaces

Since P-LAP is oxytocinase, changes in P-LAP activity in pregnancy blood just before labor onset are quite interesting for understanding the mechanisms of the onset of labor. Prior to this matter, understanding the relationship between P-LAP and progesterone is also essential. After ovulation, the human endometrium actively secretes glycoproteins and nutrients, such as peptides and amino acids, into the endometrial cavity. P-LAP localized in the lysosomes of the endometrial epithelial cells is also secreted into extracellular space. Progesterone emerges shortly after ovulation and increases the permeability of the plasma and lysosomal membranes. Progesterone is not only involved in the influx of blood-derived glucose (probably with other nutrients) into endometrial epithelial cells but also in the exocytosis of lysosomes, which transports P-LAP and other aminopeptidases and nutrients into the endometrial cavity, facilitating the fertilization of the ovum [28]. The exocytosis observed in this process is a drastic phenomenon described as apocrine and holocrine secretion [29].

The sizes of lysosomes vary from 0.1 to 1.2 µm, with the large ones being more than 10 times that of the smallest. The size of lysosomes in the endometrial epithelial cells significantly increases after ovulation [29]. Extracellular vesicles (50 nm–2 µm) are released from different cell types including the human placenta [30]. Among them, exosomes are thought to be cellular ‘debris’ with an important role in intercellular communication. P-LAP is localized on the microvilli of the trophoblast and lysosomes, not only in the apical membranes but also in the extracellular vesicle [25]. Immunoelectron microscopy has revealed localization of P-LAP on the surface of apical microvilli of the syncytiotrophoblasts, and P-LAP is likely to be released from lysosomes in the villi of syncytiotrophoblasts [24], [25] with the aid of progesterone [29], [31]. We have thus suggested that serum P-LAP may be derived from the placenta not only after conversion from the membrane-spanning to a soluble form by P-LAP secretase but also after exocytosis of the lysosomes stimulated by progesterone as in the case of endometrium [29].

## 5 Onset of labor requires progesterone withdrawal: proposal by Csapo [2]

Although the essential roles of progesterone in the maintenance of pregnancy have been generally established, the mechanisms underlying suppression of its function near term to allow labor and delivery of the conceptus are still shrouded in uncertainty. The issue that has puzzled for several decades relates to the lack of withdrawal of progesterone levels in peripheral blood of pregnant women before labor onset. Progesterone withdrawal at labor onset was taken over the concept of functional progesterone withdrawal. Progesterone responsiveness requires the expression and functional competence of progesterone receptors. Thus, changes in progesterone receptor levels and functions have been studied in relation to progesterone withdrawal. However, the roles of nuclear progesterone receptors in relation to progesterone withdrawal remain an open question [32].

**Fig. 3:**
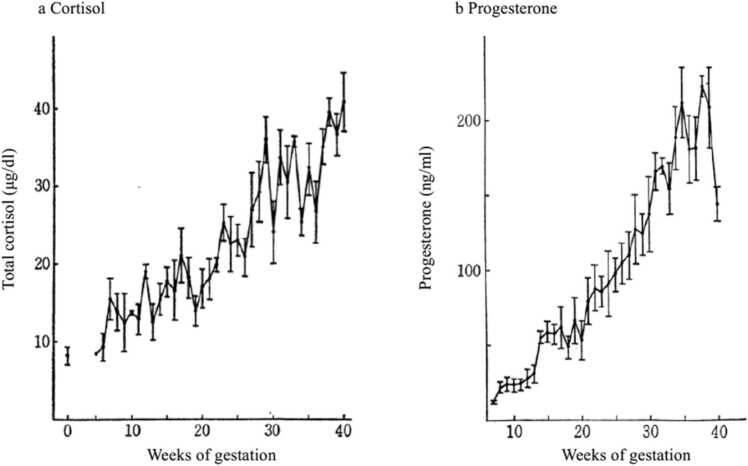
Maternal serum total cortisol (a) and progesterone (b) during normal pregnancy. Each plot is the mean ± SE. The number of pregnant patients in each plot was 3–9 (a) and 3–11(b), and the total average gestational weeks was 6.8 (a) and 7.8(b). [35].

It is therefore truly questionable why many researchers had paid little attention to the definite data of Turnbull et al. [33] showing by radioimmunoassay that labor onset in human pregnancy occurs both progesterone withdrawal and estrogen increase in plasma before labor onset in normal pregnant women. Besides this, there is only one report on progesterone withdrawal in plasma assessed by a competitive protein technique before labor onset in normal pregnant women [34]. We have also confirmed both progesterone withdrawal and cortisol increase during the last few weeks preceding labor onset by radioimmunoassay using 203 and 196 normal pregnant sera obtained randomly throughout pregnancy [35] (Fig. 3). Turnbull et al. [33] have proposed that cross-sectional studies may be of little value to estimate the changes in steroid hormones before labor onset, owing to the marked variation in individual patients.

## 6 Progesterone withdrawal reflects fetal adrenocortical maturation

Anderson et al. [36] reported that in sheep the late fall in serum progesterone levels results from enzyme induction by fetal cortisol; cortisol induces placental 17-ɑ-hydroxylase which metabolizes progesterone. Until that time, sheep placental biosynthesis of steroids is restricted to be identified mainly to those containing carbon-21 atoms because of the inactivity of the 17-ɑ-hydroxylase. When the sheep placenta is exposed to elevated levels of glucocorticoids induced by ACTH activation in the fetus, the placental 17-ɑ-hydroxylase is rapidly induced [36].

CYP17 A1 is a bifunctional enzyme with 17-ɑ-hydroxylase and 17,20-lyase activities that convert progesterone to 17OHP and then to androstenedione (A4) via the △4 pathway and pregnenolone to 17-ɑ-hydroxypregnenolone and then to DHEA through the △5 pathway (Fig. 1). Although the activity of the second reaction commits the substrate to the production of sex steroids, the first reaction is necessary for corticosteroid and mineral corticoid pathways via ACTH activation within the human placenta. Since CYP17A1 expression is significantly upregulated at term [8], the △4 pathway in the placenta is possibly activated near term in human pregnancy. Thus, progesterone withdrawal reflects fetal adrenocortical maturation.

## 7 Changes in P-LAP activity just before the onset of labor

Oxytocin acts on the uterus already primed by oxytocin at the time of parturition. Towards the term, oxytocin secretion gradually increases and reaches its peak just before labor onset [21], [22], [23]. In addition, the number of oxytocin receptors also increases in the uterine muscle near the term [37]. Due to the increased plasma level of oxytocin and increased sensitivity of the uterus to oxytocin [38], the uterus vigorously contracts, leading to the expulsion of the fetus. Therefore, oxytocin is primarily involved in the initiation and completion of human labor.

**Fig. 4:**
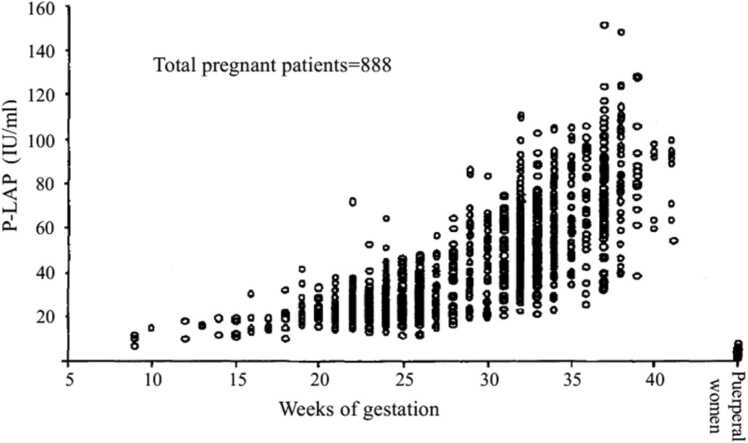
Serum P-LAP activities in healthy pregnant women (n = 888) [41].

**Fig. 5:**
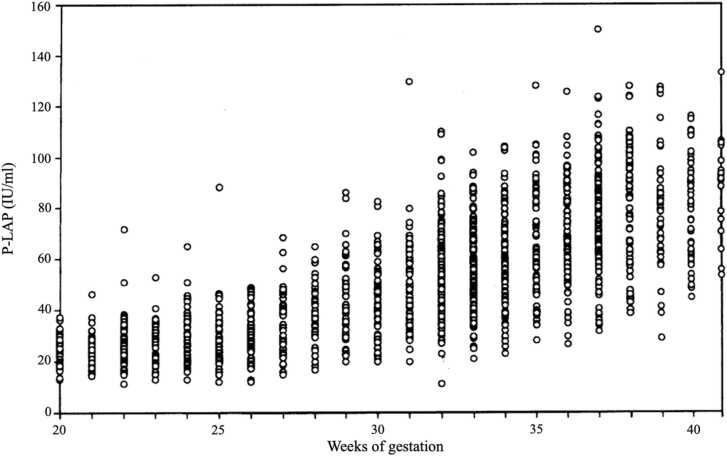
Maternal serum P-LAP activity (IU/mL) in normal pregnancy vs. gestational weeks (n = 1536) [42].

The mechanisms of the sensitivity of the uterus to oxytocin are unknown. It is acknowledged that the sensitivity of vessels to angiotensin-Ⅱ in pregnancy is due to increased angiotensinase/aminopeptidase A (APA), which degrades angiotensin-Ⅱ [39]. Since we reported that P-LAP is an oxytocin degradation enzyme [15], [26], [40], we have studied the changes in P-LAP activity during late pregnancy until normal labor onset regarding P-LAP as a regulating molecule of oxytocin sensitivity. Serum P-LAP activities were measured serially in 78 obstetrically normal pregnant women during late pregnancy [22]. The daily mean P-LAP activity progressively rises during late pregnancy, reaching a relatively high level at 13–11 days before labor onset, then slightly decreasing at 6 days before labor onset although the changes were not statistically significant and then fluctuated with minor variations [22]. Later we have studied P-LAP activities in maternal serum obtained randomly during normal pregnancy. One study using a total of 888 blood samples showed that P-LAP activities increased with advancing gestation until around 38 weeks of gestation, then the levels are flat or slightly decreased just before labor onset [41]. [Fig. 4] In another study, P-LAP activity in maternal serum increased also during pregnancy, ceasing to rising at around 38 weeks of gestation during pre-parturition using 1536 blood samples [42]. [Fig. 5] It is evident that in normal pregnancy P-LAP activities rise to around 38 weeks of gestation and no further increases in P-LAP levels during pre-parturition period from large number studies. Husselin & Leitich showed that there is a clear rise in uterine sensitivity to oxytocin 5 days before labor onset and its sensitivity is enhanced as it’s getting closer to the to the day of labor onset [38] Until labor onset, the pregnant uterus is quiescent by maintaining the balance between increasing oxytocin derived from the fetus and P-LAP in maternal blood. Thus, no further increase in P-LAP levels during 5 days just before labor onset might be meaningful, since P-LAP is no more able to keep the balance against the increased interminable fetal oxytocin with fetal maturation around labor onset.

Other studies failed to find any significant changes in serum oxytocinase levels before labor onset [43], [44]. Few reports have focused on oxytocinase level in pregnancy blood, except for a study focusing on the effect of obesity in human pregnancy [45].

**Fig. 6:**
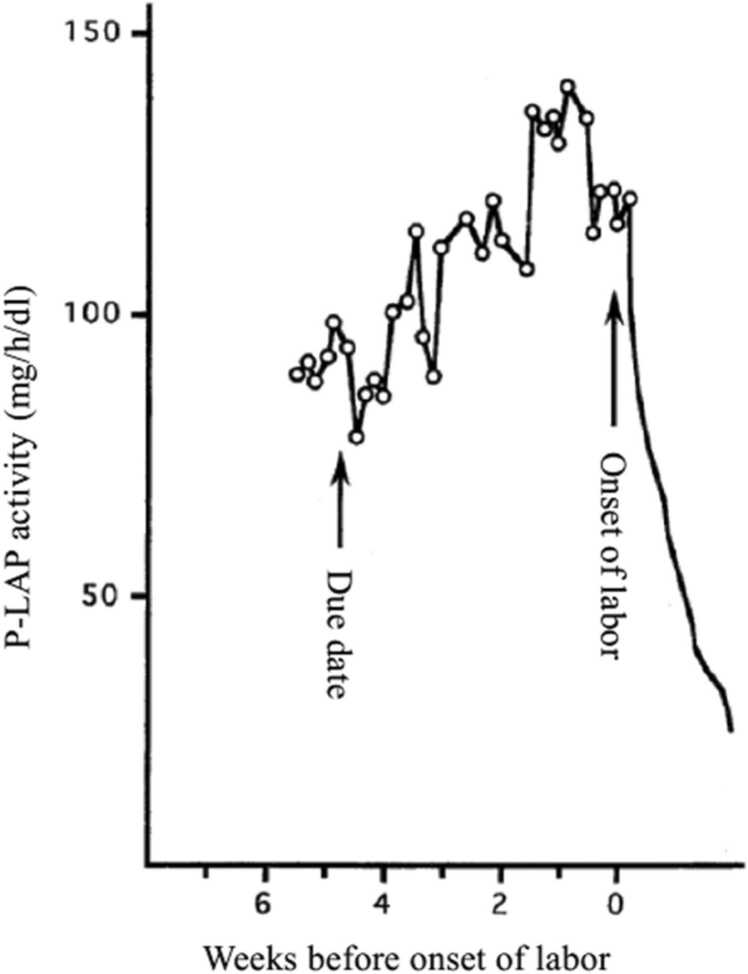
Changes in P-LAP activity in maternal blood before onset of labor in the case of the unknown due date [46].

Fig. 6 shows the changes in serum P-LAP levels during late pregnancy in one pregnant woman whose menstrual cycle was irregular. As these data were collected many years ago, ultrasonography was not available to determine the expected confinement date in Japan. Therefore, the due date was estimated according to her last menstrual period. P-LAP levels continued to increase for approximately 3 weeks after her due date and then ceased to increase during the last week. Finally, onset of labor began about five weeks after her due date and a healthy baby was born [46].

Recently, we have reported the presence of P-LAP in human urine and the importance of urinary P-LAP as an ovarian cancer biomarker [47]. In addition, we found that the changes in urinary P-LAP activity during pregnancy were essentially similar to those in maternal blood. We found an almost similar tendency of P-LAP change in the urine just before labor onset in normal pregnancy (unpublished observation). While Husslein and Leitich [38] reported a clear rise in uterine sensitivity to oxytocin during the last few days approaching labor onset, the change of P-LAP level might reflect the oxytocin sensitivity to uterus just before labor onset.

## 8 Physiological breakdown of oxytocin and P-LAP triggers the onset of labor

Until labor onset, the pregnant uterus is quiescent by maintaining the balance between increasing oxytocin derived from the fetus and P-LAP in maternal blood. Namely, the normal development of feto-placental unit contributes to the maintenance of this balance, while both fetal oxytocin and maternal P-LAP increase in parallel with the advancement of normal gestation. However, fetal maturation finally results in the physiological breakdown of the balance; although the increased interminable fetal oxytocin occurs, the progesterone withdrawal limits P-LAP release into maternal blood from the placenta just before labor onset.

The maintenance of normal uterine cervix i.e. blocking uterine cervix from normal into ripened state is essential in maintaining uterine stability during pregnancy. By the mid-1980s, prostaglandins had become established as the most effective pharmacological agent for inducing labor when the cervix is unripe [48]. A labor onset is synchronized with cervical ripening (high Bishop score), but the usefulness of prostaglandins in unripen cervix for labor induction may indicate that the changes of prostaglandins are not primarily involved in labor onset. On the other hand, the induction effects of oxytocin administration on labor are dependent upon the ripening of the uterine cervix (high Bishop score). While small-dose oxytocin is effective for the onset of labor when the cervix is ripe, a very high dose of oxytocin is required for labor induction with longer induction times in the second trimester of pregnant women [49]. These results all suggested that the prostaglandins are not primal biological molecules for labor onset.

## 9 Conclusions

Parturition is the fetal physiological phenomenon accompanied with fetal maturation. In all senses, the fetus necessarily takes the initiative in labor onset. We have acknowledged the old and pioneering works supporting this concept. Firstly, fetal hypothalamic-pituitary-adrenal axis maturation was proposed by Liggins and his colleagues [1]. Secondly, the contribution of fetal oxytocin was established by Chard et al. [12]. The former is fetal anterior pituitary maturation and the latter is fetal posterior pituitary development. The remaining one is progesterone withdrawal founded by Csapo [2]. This observation was guaranteed by the recent findings on progesterone metabolism by CYP17A1 in the human placenta. Until labor onset, pregnant uterus is probably quiescent due to the delicate balance between increasing fetal oxytocin and P-LAP in maternal blood under the control of progesterone. The contribution of fetal oxytocin to labor onset has been well acknowledged through the recognition that the effect of oxytocin in the blood is strictly regulated by its degradation by P-LAP. It becomes clear that the endocrine system plays an important role in maintenance of uterine quiescence during pregnancy and in initiating uterine activity at labor [50]. Recent studies have revealed that development and /or function of the fetal adrenal cortex may be regulated by a panoply of molecules, including transcription factors, extracellular matrix components, locally produced growth factors, and placental corticotropin releasing hormone, in addition to the primary regulator, fetal pituitary ACTH [51].

In this review, we would give brightens the clarification of mechanisms of premature delivery and the possibility of the ideal drug for the treatment of preterm labor. The pioneering works on labor onset have stood the test of time. The essential factor controlling the hierarchy of labor onset is fetal maturation itself.

## Notes

DHEA: Dehydroepiandrosterone.

CYP17A1/CytochromeP450c17:17α-hydroxylase/17,20 lyase/17,20 desmolase.

17ɑ OHP:17ɑ-hydrooxyprogesterone.

P-LAP/IRAP: Placental leucine aminopeptidase/insulin-regulated membrane aminopeptidase/ oxytocinase.

Adrenocorticotropin: ACTH.

## CRediT authorship contribution statement

Conceptualization, M.Y. and S.M.; validation, K.M., Y.K., and Y.M.; writing—original draft preparation, M.Y. and S.M.; writing—review and editing, K.M., Y.K., Y.M., H.A., S.I., S.T., H.M., T.K., E.M., K.S., and H.K.; supervision, H.K.; project administration, S.M. All authors have read and agreed to the published version of the manuscript.

## Declaration of Competing Interest

The authors declare that they have no known competing financial interests or personal relationships that could have appeared to influence the work reported in this article.
